# 2,2,6,6-Tetra­bromo-3,4,4,5-tetra­meth­oxy­cyclo­hexa­none

**DOI:** 10.1107/S160053681401472X

**Published:** 2014-06-25

**Authors:** Md. Serajul Haque Faizi, Ashraf Mashrai, M. Shahid

**Affiliations:** a Department of Chemistry, Indian Institute of Technology Kanpur, Kanpur, UP 208 016, India; b Department of Chemistry, Aligarh Muslim University, Aligarh 202 002, India

**Keywords:** crystal structure

## Abstract

In the title compound, C_10_H_14_Br_4_O_5_, synthesized from the meth­oxy Schiff base *N*-(pyridin-2-ylmeth­yl)meth­oxy­aniline and mol­ecular bromine, the cyclo­hexa­none ring has a chair conformation with one of the four meth­oxy groups equatorially orientated with respect to the carbonyl group and the others axially orientated. The C—Br bond lengthsvary from 1.942 (4) to1.964 (4) Å. In the crystal, weak C—H⋯O_carbon­yl_ hydrogen-bonding inter­actions generate chains extending along the *b*-axis direction. Also present in the structure are two short inter­molecular Br⋯O_meth­oxy_ inter­actions [3.020 (3) and 3.073 (4) Å].

## Related literature   

For the synthesis and applications of 2,2,6,6-tetra­bromo-3,4,4, 5-tetra­meth­oxy­cyclo­hexa­none and related structures, see: Khan *et al.* (2004 ▶). For applications of brominated compounds, see: Alaee (2003 ▶); Czerski & Szymanska (2005 ▶); Cupples *et al.* (2005 ▶).
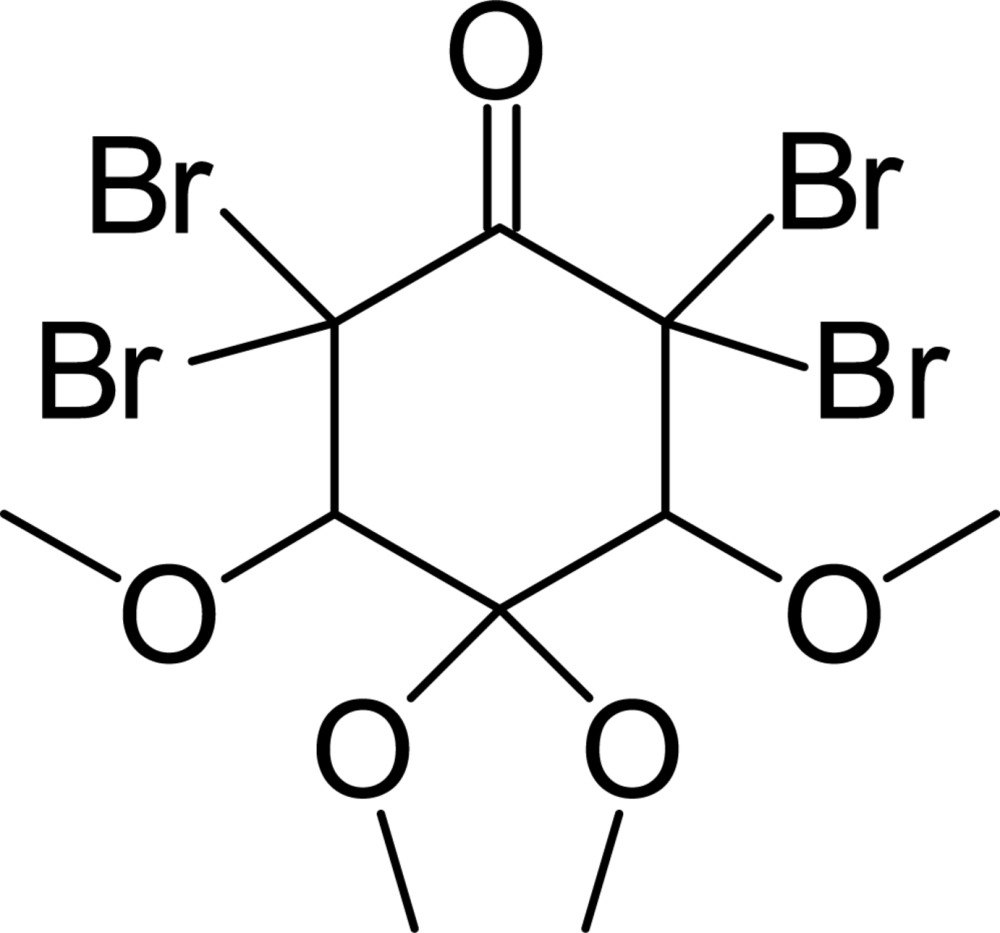


## Experimental   

### 

#### Crystal data  

C_10_H_14_Br_4_O_5_
*M* *_r_* = 533.81
Monoclinic, 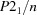

*a* = 10.396 (5) Å
*b* = 12.441 (5) Å
*c* = 12.316 (5) Å
β = 105.502 (5)°
*V* = 1535.0 (11) Å^3^
*Z* = 4
Mo *K*α radiation
μ = 10.50 mm^−1^
*T* = 100 K
0.20 × 0.15 × 0.12 mm

#### Data collection  

Bruker SMART APEX CCD diffractometer
Absorption correction: multi-scan (*SADABS*; Sheldrick, 2004 ▶) *T*_min_ = 0.228, *T*_max_ = 0.366
20265 measured reflections
2867 independent reflections
2466 reflections with *I* > 2σ(*I*)
*R*_int_ = 0.054

#### Refinement  

*R*[*F* ^2^ > 2σ(*F* ^2^)] = 0.033
*wR*(*F* ^2^) = 0.086
*S* = 1.05
2867 reflections
172 parameters
H-atom parameters constrained
Δρ_max_ = 0.85 e Å^−3^
Δρ_min_ = −1.01 e Å^−3^

### 

Data collection: *SMART* (Bruker, 2003 ▶); cell refinement: *SAINT* (Bruker, 2003 ▶); data reduction: *SAINT*; program(s) used to solve structure: *SIR97* (Altomare *et al.*, 1999 ▶); program(s) used to refine structure: *SHELXL97* (Sheldrick, 2008 ▶); molecular graphics: *DIAMOND* (Brandenberg & Putz, 2006 ▶); software used to prepare material for publication: *DIAMOND*.

## Supplementary Material

Crystal structure: contains datablock(s) global, I. DOI: 10.1107/S160053681401472X/zs2305sup1.cif

Structure factors: contains datablock(s) I. DOI: 10.1107/S160053681401472X/zs2305Isup2.hkl

Supporting information file. DOI: 10.1107/S160053681401472X/zs2305Isup3.cml

CCDC reference: 1009490

Additional supporting information:  crystallographic information; 3D view; checkCIF report

## Figures and Tables

**Table 1:** Hydrogen-bond geometry (Å, °)

*D*—H⋯*A*	*D*—H	H⋯*A*	*D*⋯*A*	*D*—H⋯*A*
C3—H3⋯O1^i^	0.98	2.41	3.361 (5)	163

Symmetry code: (i) 
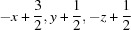
.

**Fig. 1.**
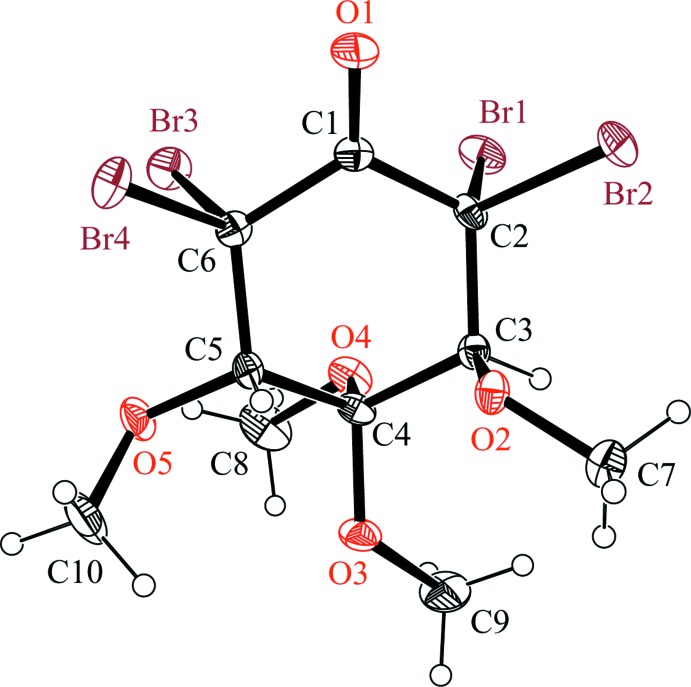
The molecular conformation and atom-numbering scheme for the title compound, with non-H atoms drawn as 40% probability displacement ellipsoids.

**Fig. 2.**
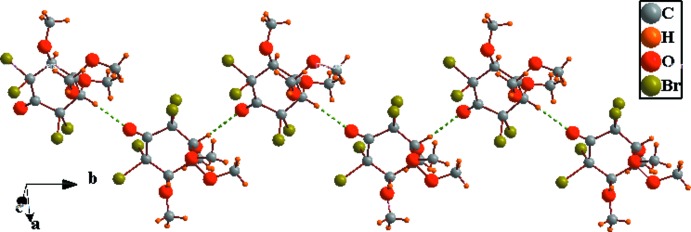
The one-dimensional hydrogen-bonded chain structure in the title compound extending along b, with hydrogen bonds shown as dashed lines.

**Fig. 3.**
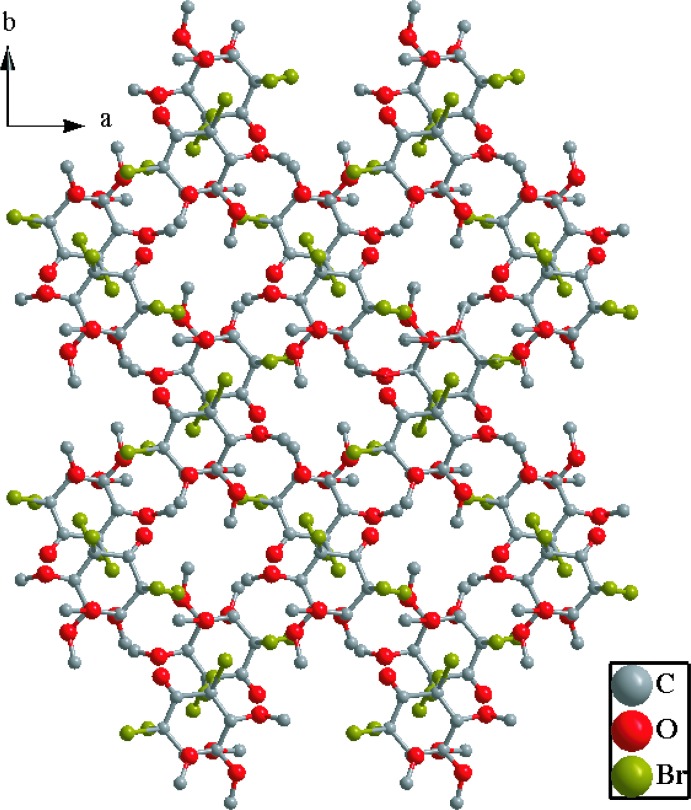
The molecular packing viewed along the c-axial direction.

## supplementary crystallographic information

### S1. Comment

Brominated organic compounds have a broad spectrum of applications. The polybrominated biphenyls, polybrominated diphenylethers and hexabromobenzene are widely used as flame retardants. Other brominated molecules such as 1,4-dibromobenzene (1,4-DBB) and bromoxynil serve as fumigants, as intermediates in the synthesis of dyes, as agrochemicals, pharmaceuticals, or herbicides (Alaee *et al.*, 2003; Czerski *et al.*, 2005; Cupples *et al.*, 2005). Very few examples of the synthesis and applications of compounds similar to the title compound, C_10_H_14_Br_4_O_5_, 2,2,6,6-tetrabromo-3,4,4,5-tetramethoxycyclohexanone (TBTM) have been reported in the literature (Khan *et al.*, 2004). Our synthesis of this compound, by the reaction of the Schiff base *N*-(pyridin-2-ylmethyl)methoxyaniline (PMMA) with molecular bromine in methanol has not previously been reported and its structure is reported herein.

In the racemic title compound (Fig. 1), the cyclohexanone ring has a chair conformation with one of the four methoxy groups equatorially oriented with respect to the carbonyl group and the others axially orientated. The C—Br bond lengths are variable [C—Br = 1.946 (4), 1.964 (4),1.942 (4) and 1.959 (4) Å]. In the crystal, weak intermolecular C—H···O_carbonyl_ hydrogen-bonding interactions (Table 1) generate one-dimensional chains extending along *b* (Fig. 2). Also present in the structure are a number of short intramoleculat Br···O contacts [Br···O, 2.961 (3)–3.169 (4) Å] as well as two short intermolecular Br···O_methoxy_ interactions [Br4···O2^ii^, 3.020 (3) Å and Br1···O3^iii^, 3.073 (4) Å] [for symmetry codes: (ii) *x* + 1/2, -*y* + 1/2, *z* + 1/2; (iii) -*x*, -*y* + 1, -*z*]. The overall packing in the unit cell is shown in Fig. 3.

### S2. Experimental

Molecular bromine (0.15 g, 1.00 mmol) was added carefully to a methanolic solution (10 mL) of *N*-(pyridin-2-ylmethyl)methoxyaniline (0.20 g, 1.00 mmol). The color of the reaction mixture turned immediately from yellow to red and a yellow precipitate formed after one hour of stirring. The precipitate was filtered off, washed first with acetone then with diethylether and then redissolved in deuterated methanol and kept in an NMR tube for crystallization. Crystals of the title compound suitable for X-ray analysis was obtained within 15 days by slow evaporation of the solvent.

### S3. Refinement

All H-atoms were positioned geometrically and refined using a riding model with C—H = 0.98 ° (methylene) or 0.96 Å (methyl) and *U*_iso_(H) = 1.2 or 1.5*U*_eq_(C).

### Crystal data

**Table:**

C_10_H_14_Br_4_O_5_	*F*(000) = 1016
*M**_r_* = 533.81	*D*_x_ = 2.310 Mg m^−^^3^
Monoclinic, *P*2_1_/*n*	Mo *K*α radiation, λ = 0.71073 Å
Hall symbol: -P 2yn	Cell parameters from 999 reflections
*a* = 10.396 (5) Å	θ = 2.6–28.6°
*b* = 12.441 (5) Å	µ = 10.50 mm^−^^1^
*c* = 12.316 (5) Å	*T* = 100 K
β = 105.502 (5)°	Block, yellow
*V* = 1535.0 (11) Å^3^	0.20 × 0.15 × 0.12 mm
*Z* = 4	

### Data collection

**Table:**

Bruker SMART APEX CCD diffractometer	2867 independent reflections
Radiation source: fine-focus sealed tube	2466 reflections with *I* > 2σ(*I*)
Graphite monochromator	*R*_int_ = 0.054
ω scans	θ_max_ = 25.5°, θ_min_ = 2.3°
Absorption correction: multi-scan (*SADABS*; Sheldrick, 2004)	*h* = −12→12
*T*_min_ = 0.228, *T*_max_ = 0.366	*k* = −15→15
20265 measured reflections	*l* = −14→14

### Refinement

**Table:**

Refinement on *F*^2^	Primary atom site location: structure-invariant direct methods
Least-squares matrix: full	Secondary atom site location: difference Fourier map
*R*[*F*^2^ > 2σ(*F*^2^)] = 0.033	Hydrogen site location: inferred from neighbouring sites
*wR*(*F*^2^) = 0.086	H-atom parameters constrained
*S* = 1.05	*w* = 1/[σ^2^(*F*_o_^2^) + (0.0476*P*)^2^ + 1.8635*P*] where *P* = (*F*_o_^2^ + 2*F*_c_^2^)/3
2867 reflections	(Δ/σ)_max_ = 0.001
172 parameters	Δρ_max_ = 0.85 e Å^−^^3^
0 restraints	Δρ_min_ = −1.01 e Å^−^^3^

### Special details

**Table:**

Geometry. All esds (except the esd in the dihedral angle between two l.s. planes) are estimated using the full covariance matrix. The cell esds are taken into account individually in the estimation of esds in distances, angles and torsion angles; correlations between esds in cell parameters are only used when they are defined by crystal symmetry. An approximate (isotropic) treatment of cell esds is used for estimating esds involving l.s. planes.
Refinement. Refinement of F^2^ against ALL reflections. The weighted R-factor wR and goodness of fit S are based on F^2^, conventional R-factors R are based on F, with F set to zero for negative F^2^. The threshold expression of F^2^ > 2sigma(F^2^) is used only for calculating R-factors(gt) etc. and is not relevant to the choice of reflections for refinement. R-factors based on F^2^ are statistically about twice as large as those based on F, and R- factors based on ALL data will be even larger.

### Fractional atomic coordinates and isotropic or equivalent isotropic displacement parameters (Å^2^)

**Table:**

	*x*	*y*	*z*	*U*_iso_*/*U*_eq_	
C1	0.6442 (4)	0.0403 (3)	0.2171 (3)	0.0253 (8)	
C2	0.7031 (4)	0.1525 (3)	0.2524 (3)	0.0244 (8)	
C3	0.6061 (4)	0.2460 (3)	0.2078 (3)	0.0236 (8)	
H3	0.6444	0.3136	0.2429	0.028*	
C4	0.4661 (4)	0.2290 (3)	0.2259 (3)	0.0253 (8)	
C5	0.4051 (4)	0.1254 (3)	0.1655 (3)	0.0231 (8)	
H5	0.3972	0.1352	0.0850	0.028*	
C6	0.4933 (4)	0.0256 (3)	0.2045 (3)	0.0245 (8)	
C7	0.6279 (5)	0.3481 (3)	0.0472 (4)	0.0381 (11)	
H7A	0.6112	0.3431	−0.0331	0.057*	
H7B	0.7215	0.3594	0.0805	0.057*	
H7C	0.5784	0.4073	0.0657	0.057*	
C8	0.3675 (5)	0.2322 (5)	0.3844 (4)	0.0517 (14)	
H8A	0.3942	0.2343	0.4652	0.078*	
H8B	0.3162	0.1683	0.3597	0.078*	
H8C	0.3141	0.2943	0.3561	0.078*	
C9	0.4060 (5)	0.4176 (4)	0.2142 (5)	0.0489 (13)	
H9A	0.3407	0.4663	0.1706	0.073*	
H9B	0.4933	0.4387	0.2097	0.073*	
H9C	0.4029	0.4193	0.2914	0.073*	
C10	0.1701 (5)	0.1231 (5)	0.0796 (5)	0.0619 (17)	
H10A	0.0869	0.1052	0.0951	0.093*	
H10B	0.1814	0.0800	0.0182	0.093*	
H10C	0.1699	0.1978	0.0599	0.093*	
O1	0.7121 (3)	−0.0326 (2)	0.2003 (3)	0.0409 (8)	
O2	0.5874 (3)	0.2513 (2)	0.0895 (2)	0.0273 (6)	
O3	0.3779 (3)	0.3107 (2)	0.1706 (3)	0.0322 (7)	
O4	0.4841 (3)	0.2319 (3)	0.3426 (2)	0.0336 (7)	
O5	0.2763 (3)	0.1030 (2)	0.1766 (3)	0.0346 (7)	
Br1	0.76975 (4)	0.15408 (4)	0.41548 (4)	0.03859 (15)	
Br2	0.86086 (4)	0.16954 (4)	0.19556 (5)	0.04340 (15)	
Br3	0.48249 (5)	−0.02879 (4)	0.35148 (4)	0.03964 (15)	
Br4	0.42888 (5)	−0.08868 (3)	0.09570 (4)	0.03854 (14)	

### Atomic displacement parameters (Å^2^)

**Table:**

	*U*^11^	*U*^22^	*U*^33^	*U*^12^	*U*^13^	*U*^23^
C1	0.028 (2)	0.028 (2)	0.0225 (19)	0.0045 (17)	0.0103 (16)	0.0020 (16)
C2	0.0189 (19)	0.031 (2)	0.026 (2)	0.0010 (15)	0.0107 (16)	−0.0048 (16)
C3	0.0242 (19)	0.0233 (19)	0.0241 (19)	−0.0026 (15)	0.0077 (16)	−0.0036 (15)
C4	0.0220 (19)	0.032 (2)	0.0231 (19)	0.0070 (16)	0.0088 (16)	−0.0032 (17)
C5	0.0210 (19)	0.029 (2)	0.0227 (19)	−0.0029 (16)	0.0120 (16)	0.0013 (16)
C6	0.028 (2)	0.026 (2)	0.0216 (18)	0.0004 (16)	0.0100 (16)	0.0013 (15)
C7	0.042 (3)	0.034 (2)	0.040 (3)	−0.0062 (19)	0.014 (2)	0.004 (2)
C8	0.044 (3)	0.079 (4)	0.041 (3)	0.012 (3)	0.028 (2)	−0.008 (3)
C9	0.051 (3)	0.031 (3)	0.065 (3)	0.013 (2)	0.016 (3)	−0.014 (2)
C10	0.020 (2)	0.092 (4)	0.067 (4)	−0.012 (3)	−0.001 (2)	0.032 (3)
O1	0.0357 (17)	0.0313 (17)	0.058 (2)	0.0098 (13)	0.0158 (15)	−0.0045 (15)
O2	0.0300 (15)	0.0288 (15)	0.0262 (14)	−0.0063 (12)	0.0129 (12)	−0.0037 (11)
O3	0.0279 (15)	0.0296 (15)	0.0391 (16)	0.0100 (12)	0.0092 (13)	−0.0027 (13)
O4	0.0319 (16)	0.0483 (18)	0.0256 (15)	0.0072 (13)	0.0161 (13)	−0.0052 (13)
O5	0.0206 (14)	0.0475 (19)	0.0388 (17)	−0.0042 (12)	0.0134 (13)	0.0066 (14)
Br1	0.0321 (2)	0.0495 (3)	0.0289 (2)	0.00714 (19)	−0.00106 (18)	−0.00593 (19)
Br2	0.0262 (2)	0.0488 (3)	0.0627 (3)	0.00206 (18)	0.0249 (2)	0.0017 (2)
Br3	0.0440 (3)	0.0459 (3)	0.0336 (2)	0.0030 (2)	0.0184 (2)	0.0132 (2)
Br4	0.0484 (3)	0.0277 (2)	0.0401 (3)	−0.01180 (18)	0.0127 (2)	−0.00577 (18)

### Geometric parameters (Å, º)

**Table:**

C1—O1	1.201 (5)	C7—O2	1.420 (5)
C1—C2	1.540 (6)	C7—H7A	0.9600
C1—C6	1.545 (5)	C7—H7B	0.9600
C2—C3	1.540 (5)	C7—H7C	0.9600
C2—Br1	1.942 (4)	C8—O4	1.439 (5)
C2—Br2	1.959 (4)	C8—H8A	0.9600
C3—O2	1.419 (5)	C8—H8B	0.9600
C3—C4	1.545 (5)	C8—H8C	0.9600
C3—H3	0.9800	C9—O3	1.434 (5)
C4—O4	1.399 (5)	C9—H9A	0.9600
C4—O3	1.416 (5)	C9—H9B	0.9600
C4—C5	1.537 (6)	C9—H9C	0.9600
C5—O5	1.410 (5)	C10—O5	1.415 (6)
C5—C6	1.542 (5)	C10—H10A	0.9600
C5—H5	0.9800	C10—H10B	0.9600
C6—Br4	1.946 (4)	C10—H10C	0.9600
C6—Br3	1.964 (4)		
			
O1—C1—C2	121.7 (4)	C1—C6—Br3	104.8 (2)
O1—C1—C6	121.4 (4)	Br4—C6—Br3	106.80 (19)
C2—C1—C6	116.9 (3)	O2—C7—H7A	109.5
C1—C2—C3	114.2 (3)	O2—C7—H7B	109.5
C1—C2—Br1	107.8 (3)	H7A—C7—H7B	109.5
C3—C2—Br1	112.3 (3)	O2—C7—H7C	109.5
C1—C2—Br2	107.7 (3)	H7A—C7—H7C	109.5
C3—C2—Br2	108.8 (3)	H7B—C7—H7C	109.5
Br1—C2—Br2	105.50 (18)	O4—C8—H8A	109.5
O2—C3—C2	107.3 (3)	O4—C8—H8B	109.5
O2—C3—C4	106.2 (3)	H8A—C8—H8B	109.5
C2—C3—C4	113.4 (3)	O4—C8—H8C	109.5
O2—C3—H3	109.9	H8A—C8—H8C	109.5
C2—C3—H3	109.9	H8B—C8—H8C	109.5
C4—C3—H3	109.9	O3—C9—H9A	109.5
O4—C4—O3	111.5 (3)	O3—C9—H9B	109.5
O4—C4—C5	116.4 (3)	H9A—C9—H9B	109.5
O3—C4—C5	103.8 (3)	O3—C9—H9C	109.5
O4—C4—C3	105.8 (3)	H9A—C9—H9C	109.5
O3—C4—C3	110.2 (3)	H9B—C9—H9C	109.5
C5—C4—C3	109.1 (3)	O5—C10—H10A	109.5
O5—C5—C4	113.5 (3)	O5—C10—H10B	109.5
O5—C5—C6	108.2 (3)	H10A—C10—H10B	109.5
C4—C5—C6	112.9 (3)	O5—C10—H10C	109.5
O5—C5—H5	107.3	H10A—C10—H10C	109.5
C4—C5—H5	107.3	H10B—C10—H10C	109.5
C6—C5—H5	107.3	C3—O2—C7	116.3 (3)
C5—C6—C1	116.0 (3)	C4—O3—C9	116.4 (3)
C5—C6—Br4	107.9 (2)	C4—O4—C8	118.2 (3)
C1—C6—Br4	108.0 (3)	C5—O5—C10	115.5 (3)
C5—C6—Br3	112.9 (3)		
			
O1—C1—C2—C3	147.2 (4)	C3—C4—C5—C6	56.7 (4)
C6—C1—C2—C3	−33.0 (5)	O5—C5—C6—C1	−170.4 (3)
O1—C1—C2—Br1	−87.3 (4)	C4—C5—C6—C1	−44.0 (4)
C6—C1—C2—Br1	92.6 (3)	O5—C5—C6—Br4	68.3 (3)
O1—C1—C2—Br2	26.2 (5)	C4—C5—C6—Br4	−165.3 (3)
C6—C1—C2—Br2	−154.0 (3)	O5—C5—C6—Br3	−49.5 (4)
C1—C2—C3—O2	−69.5 (4)	C4—C5—C6—Br3	77.0 (3)
Br1—C2—C3—O2	167.3 (2)	O1—C1—C6—C5	−148.5 (4)
Br2—C2—C3—O2	50.9 (3)	C2—C1—C6—C5	31.7 (5)
C1—C2—C3—C4	47.5 (4)	O1—C1—C6—Br4	−27.3 (4)
Br1—C2—C3—C4	−75.7 (3)	C2—C1—C6—Br4	152.9 (3)
Br2—C2—C3—C4	167.9 (2)	O1—C1—C6—Br3	86.3 (4)
O2—C3—C4—O4	−175.7 (3)	C2—C1—C6—Br3	−93.5 (3)
C2—C3—C4—O4	66.7 (4)	C2—C3—O2—C7	−118.5 (4)
O2—C3—C4—O3	−55.0 (4)	C4—C3—O2—C7	119.9 (4)
C2—C3—C4—O3	−172.6 (3)	O4—C4—O3—C9	50.5 (5)
O2—C3—C4—C5	58.4 (4)	C5—C4—O3—C9	176.6 (4)
C2—C3—C4—C5	−59.2 (4)	C3—C4—O3—C9	−66.7 (5)
O4—C4—C5—O5	60.8 (4)	O3—C4—O4—C8	52.1 (5)
O3—C4—C5—O5	−62.2 (4)	C5—C4—O4—C8	−66.7 (5)
C3—C4—C5—O5	−179.7 (3)	C3—C4—O4—C8	172.0 (4)
O4—C4—C5—C6	−62.8 (4)	C4—C5—O5—C10	105.2 (5)
O3—C4—C5—C6	174.3 (3)	C6—C5—O5—C10	−128.7 (4)

### Hydrogen-bond geometry (Å, º)

**Table:**

*D*—H···*A*	*D*—H	H···*A*	*D*···*A*	*D*—H···*A*
C3—H3···O1^i^	0.98	2.41	3.361 (5)	163

Symmetry code: (i) −*x*+3/2, *y*+1/2, −*z*+1/2.
